# One-year incidence of depression, anxiety, or stress disorders following a first-time heart failure diagnosis: A Danish nationwide registry-based study

**DOI:** 10.1016/j.ahjo.2022.100240

**Published:** 2022-12-08

**Authors:** M.W. Pedersen, R. Rørth, M.P. Andersen, M. Sessa, C. Polcwiartek, S.J. Riddersholm, G. Gislason, S.L. Kristensen, N.H. Andersen, L. Køber, P. Søgaard, C. Torp-Pedersen, K.H. Kragholm

**Affiliations:** a Department of Cardiology, Aalborg University Hospital, Hobrovej18-22, 9000 Aalborg, Denmark; b Department of Cardiology, Rigshospitalet, Copenhagen University Hospital, Blegdamsvej 9, 2100 Copenhagen, Denmark; c Department of Clinical Research, Nordsjællands Hospital, Dyrehavevej 29, 3400 Hillerød, Denmark; d Department of Drug Design and Pharmacology, University of Copenhagen, Universitetsparken 2, 2100 Copenhagen, Denmark; e Department of Cardiology, Copenhagen University Hospital Herlev and Gentofte, Hospitalsvej 1, 2900 Hellerup, Denmark; f The Danish Heart Foundation, Vognmagergade 7,3, 1120 Copenhagen K, Denmark; g Unit of Clinical Biostatistics and Epidemiology, Aalborg University Hospital, Sdr. Skovvej 15, 9000 Aalborg, Denmark

**Keywords:** Heart disease, Affective disorders, Antidepressants, Epidemiology, Population studies

## Abstract

**Study objective:**

To examine first-time depression, anxiety, stress disorders or psychotropic drug prescriptions within one year after incident heart failure (HF).

**Design:**

Nationwide Epidemiological registry study.

**Setting:**

National patient registries.

**Participants:**

Patients in Denmark with a first-time HF diagnosis during 2005–2015.

**Interventions:**

None.

**Main outcome measures:**

Incidences of depression, anxiety, stress disorders or first-time prescription of a psychotropic drug were determined.

**Results:**

A total of 94,712 HF patients and 473,560 matched controls were included (median age 74.0 [64.0–81.0] years, 60.8 % males). At one year after incident HF, 11.9 % met the primary composite endpoint (depression, anxiety, or stress disorders or prescription of related psychotropic drugs), with 8.6 % outpatients and 13.3 % in-patients, versus 2.4 % of the controls. Starting psychotropic medication accounted for most of the composite endpoint events, as 11.6 % of the HF patients started antidepressants, anxiolytics, hypnotics, or sedative drugs (2.4 % among controls), while 0.6 % received a registered diagnosis of depression, anxiety, or stress disorder (<0.1 % among controls). The relative risk of psychotropic drug prescriptions in HF patients versus controls (standardized to the age, sex, and selected comorbidity distributions of all included subjects) was 3.85 [95 % CI 3.73–3.98]. The corresponding relative risk for one of the psychiatric diagnoses was 12.90 [95 % CI 10.60–15.19].

**Conclusion:**

A substantial part of patients with newly diagnose heart failure started treatment with psychotropic drugs whereas only a small fraction was registered with depression, anxiety, or stress disorders within one-year follow-up. The incidences were significantly higher than in the background population.

## 1 Introduction

Clinically significant depression has previously been found in 21–29 % of patients with heart failure (HF) in two meta-analyses and the prevalence has remained stable over the last decade [1], [2], [3]. Depression is associated with increased mortality and hospital readmission in patients with HF and this association seems not to depend on the traditional risk factors such as deteriorated cardiac function and age [4], [5], [6]. A recent study found an association between self-reported anxiety symptoms as well as depressive symptoms and all-cause or cardiovascular mortality in patients with HF [7]. A graded association between depressive symptoms and functional decline in HF patients has also been reported and some may even develop major depression or commit suicide after being diagnosed with HF [1], [8].

Recognition of depression is challenging in patients with HF and for this reason, the current guidelines from European Society of Cardiology recommend systematic screening for depression in HF patients [9], [10]. Several studies have examined the prevalence of depression in patients with HF. However, in addition to a few small studies, only one single larger study has evaluated the incidence of depression in patients with HF in which incident depression/first-ever depression was significantly increased compared to non-HF controls [11], [12], [13]. Thus, the aim of this large-scale nationwide study was to determine the incidences and absolute and relative risks of depression, anxiety, or stress disorders or prescription of a psychotropic drug (as proxies for the abovementioned diseases) during the first year after being diagnosed with HF.

## 2 Materials and methods

### 2.1 Registries

The health care system in Denmark is tax-funded and therefore, Danish citizens have equal access to health care. All Danish citizens have a unique and permanent personal identification number (CPR-number). This number provides a unique opportunity for accurate record linkage between nationwide registries on an individual level. In the present study, seven different Danish national registries were used as data sources: 1) *The Danish Civil Registration System* [14]; 2) *The National Patient Register* which contains data on all in-patient contacts with the Danish healthcare system from 1977 and onwards and since 1995 also data on outpatient, emergency room and psychiatric patient care [15]; 3) *The Danish National prescription Register* in which individual-level data on all dispensed prescriptions are registered [16]; 4) *The Danish Register of Causes of Death* [17]; 5) *The Psychiatric Central Research Register* which contains registration of patients treated at psychiatric departments in Denmark since 1970 (outpatient contacts since 1995) [18]; 6) *The Population Educational* Register [19]; and 7) *Danish registers on personal income and transfer* payments [20].

### 2.2 Study population

By linking data from the Danish Civil Registration Register and The National Patient Register, all patients in Denmark with a first-time diagnosis of HF (in-hospital or outpatient) from 1 January 2005 to 31 December 2015 were identified. We defined HF according to the 10th and 8th revision of the International Classification of Diseases (ICD-10 and ICD-8) (see Supplemental Table 1). Patients were excluded if they had a history of mental disorder. Thus, patients who had a psychiatric diagnosis prior to the HF diagnosis (within the last 10 years) were excluded. Similarly, patients were excluded if they, within the last year before the diagnosis of HF, had collected a prescription for antipsychotic drugs, antidepressants, anxiolytics, hypnotic, or sedative drugs. The psychiatric diagnoses were retrieved from in-hospital or outpatient psychiatric contacts excluding diagnoses from psychiatric emergency room. All ICD-10 Classification diagnoses according to mental or behavioural disorders were included except the codes for Mental or behavioural disorders due to psychotropic drugs. Details are provided in Supplemental Table 2 and Supplemental Table 3 according to diagnoses and psychotropic drugs respectively.

To promote comparability between the HF population and the general population, risk set matching was used. Each patient with HF was matched 1:5 according to sex, age, and time of the HF diagnosis (year and month). Patients were excluded if it was impossible to achieve five unique matched controls.

### 2.3 Study variables

Data on age, sex, and status of living alone was retrieved from the Danish Civil Personal Registrations Registry. Data on pre-existing comorbidity was retrieved from the Danish National Patient Register. However, for diabetes, chronic obstructive pulmonary disease (COPD) and hypertension, data was also retrieved from the Danish National Prescription Register using drugs as proxies for these diseases. See Supplemental Table 4 for details on the ICD-10 - and ATC codes that were used. Income data (registered in the last year before inclusion) and data on the highest achieved educational level were attained from the Danish registers on personal income and transfer payments and the Population Educational Register, respectively. The Danish educational levels are converted to the *International Standard Classification of Education* (ISCED) from 2011, which classifies educational levels from 0 to 8 [21].

### 2.4 Outcomes

Using ICD-10 codes from in-hospital and outpatient psychiatric contacts, incident depression, anxiety, or stress disorders during the first year after the diagnosis of HF was analysed. By means of the Danish National Prescription Register, we identified the proportion of patients who had started collecting a prescription of antidepressants, anxiolytics, hypnotics, or other sedative drugs during the first year after the HF diagnosis. See Supplemental Table 5 for details about ICD - and ATC codes that were used. We have defined a primary composite endpoint. A patient or a control met the primary composite endpoint if they have achieved at least one of the above-mentioned psychiatric diagnoses or have collected a prescription of at least one of the above-mentioned medications. Secondary outcomes were the composite of the above-mentioned diagnoses and medications separately.

### 2.5 Statistics

Continuous variables are reported as median and 25–75 % percentiles, and categorical variables as counts and percentages. Cumulative incidences are reported using the Aalen-Johansen estimator. Multivariable Cox regression average treatment effect modelling was used to derive absolute and relative risks of outcomes standardized to the age, sex, and selected comorbidity distributions of all included subjects. The selected comorbidities used for standardization included previous myocardial infarction, ischemic heart disease, diabetes, hypertension, stroke, peripheral artery disease, chronic kidney disease and chronic obstructive pulmonary disease. In additional analyses, highest education level as well as living alone status were added as covariates. A two-sided *p*-value <0.05 was considered statistically significant. All analyses were performed using SAS (version 9.4, SAS Institute, Cary, NC, USA) and R statistical software (version 3.6.1).

### 2.6 Ethics

In Denmark, register-based studies conducted for the sole purpose of statistics and scientific research do not require ethical approval or informed consent by law. However, the present study is approved by the data responsible institute (The Capital Region of Denmark (Approval number: P-2019-191) in accordance with the General Data Protection Regulation (GDPR).

## 3 Results

### 3.1 Study population

**Fig. 1:**
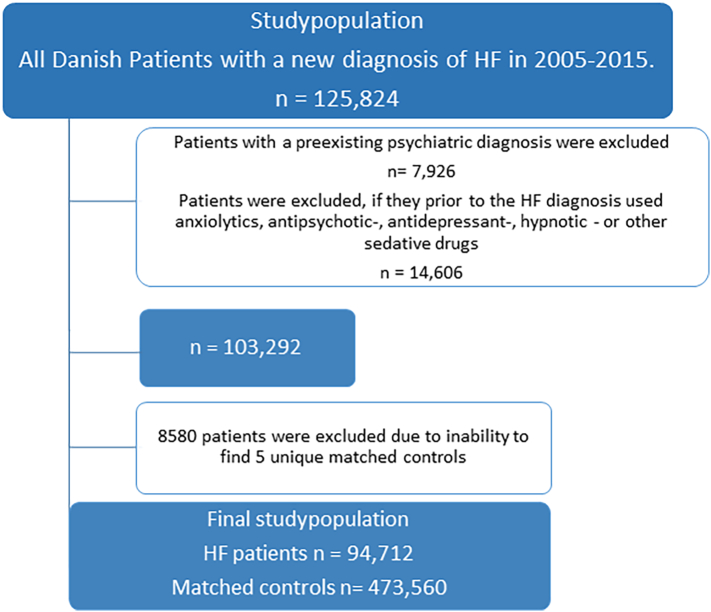
Patient selection.

During 2005–2015, 125,824 patients with a first-time diagnosis of HF were identified. A total of 7926 patients were excluded due to a pre-existing psychiatric diagnosis and 14,606 patients were excluded due to usage of anxiolytics, antipsychotic -, hypnotic -, antidepressant -, or other sedative drugs prior to the HF diagnosis. Additional 8580 patients were excluded due to inability to find matched controls. Thus, the final study population comprised 94,712 patients with HF and 473,560 controls (Fig. 1). Demographics are presented in Table 1. The median age was 74.0 [IQR 64.0, 81.0] years and 60.8 % were males. Level of income and education were lower among the patients with HF and a larger percentage of patients with HF lived alone when compared to controls (45.3 % vs 34.7 %).

**Table 1:** Characteristics of the HF patients and their matched controls.

	HF patients (*n* = 94,712)	Controls (*n* = 473,560)
Age in years, median [iqr]	74.0 [64.0, 81.0]	74.0 [64.0, 81.0]
Male gender, n (%)	57,575 (60.8)	287,875 (60.8)
Living alone, n (%)	42,325 (45.3)	116,306 (34.7)
Data missing, n	1254	138,491
Household income, median [iqr]^a^	38,284	40,700
	[28,176-56,201]	[28,076-66,340]
Data missing, n	711	112,292
Individual income, median [iqr]	226,300	237,327
	[182,273-320,713]	[174,035-365,305]
Data missing, n	711	112,292
Educational level: n (%)		
ISCED 0–2	40,490 (48.5)	126,875 (41.7)
ISCED 3	30,827 (37.0)	115,765 (38.1)
ISCED 5–6	8951 (10.7)	45,643 (15.0)
ISCED 7–8	3138 (3.8)	15,898 (5.2)
Data missing, n	11,306	169,379
Comorbidity: n (%)		
Acute myocardial infarction	12,916 (13.6)	9628 (2.0)
Ischemic heart disease	30,096 (31.8)	29,053 (6.1)
Diabetes	14,298 (15.1)	16,887 (3.6)
Hypertension	56,876 (60.1)	121,665 (25.7)
Stroke	10,088 (10.7)	14,293 (3.0)
Peripheral artery disease	8849 (9.3)	8506 (1.8)
Chronic kidney disease	5189 (5.5)	3975 (0.8)
COPD	12,223 (12.9)	10,372 (2.2)

a Euro.

### 3.2 Outcomes

**Fig. 2:**
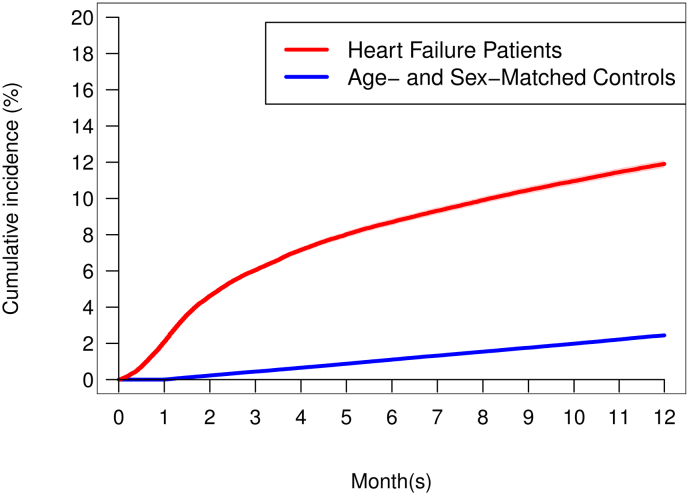
One-year cumulative incidence of composite endpoint in HF patients versus age- and sex-matched controls. Composite endpoint: a first-time registered diagnosis of depression, anxiety, or stress disorder or first-time prescription of related psychotropic drugs.

**Fig. 3:**
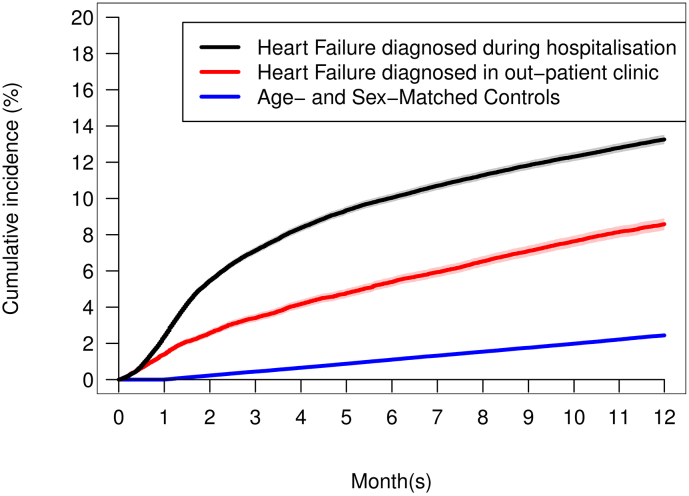
One-year cumulative incidence of composite endpoint in HF patients divided by whether the HF diagnosis was made in an in- or outpatient clinic versus age- and sex-matched controls. Composite endpoint: a first-time registered diagnosis of depression, anxiety, or stress disorder or first-time prescription of related psychotropic drugs.

Crude one-year outcomes are presented in Table 2, and Fig. 2 shows the cumulative incidence of the composite endpoint for patients with HF and controls. During the first year after the HF diagnosis, 11.9 % of the patients met the primary composite endpoint (achieved one of the psychiatric diagnoses or started collecting prescriptions for psychotropic medication), while was the case for 2.4 % of the controls. Dividing the patients in those who were diagnosed with HF during hospitalisation and those diagnosed in out-patient clinic settings we found that the incidence of the composite endpoint was lower in the out-patient group but was still significantly higher compared to the control group, with 8.6 % of outpatients, 13.3 % of in-patients and 2.4 % of controls (Fig. 3).

**Table 2:** Development of depression, anxiety and stress disorder in HF patients and their matched controls during the first year after the HF diagnosis.

	HF-patients (n = 94,712)	Controls (n = 473,560)	p-Value
Depression (ICD-10)^a^	305 (0.3)	156 (<0.1)	<0.0001
Anxiety (ICD-10^a^	56 (0.1)	14 (<0.1)	<0.0001
Stress disorder (ICD-10)^a^	238 (0.3)	66 (<0.1)	<0.0001
Antidepressants^a^	4592 (4.8)	5076 (1.1)	<0.0001
Anxiolytics^a^	2816 (3.0)	3266 (0.7)	<0.0001
Hypnotics^a^	4748 (5.0)	4819 (1.0)	<0.0001
Other sedative drugs^a^	701 (0.7)	390 (0.1)	<0.0001
Primary composite endpoint^b^	11,279 (11.9)	11,569 (2.4)	<0.0001
Composite of any of the abovementioned diagnoses	525 (0.6)	209 (<0.1)	<0.0001
Composite of any of the abovementioned drugs	11,004 (11.6)	11.525 (2.4)	<0.0001
Death^c^	17,291 (18.3)	8694 (1.8)	<0.0001

Data are expressed as numbers (%).

a Cumulative incidence (Aalen-Johansen estimates).

b Development of either depression, anxiety or stress disorder or started with antidepressants, anxiolytics, hypnotics, or other sedative drugs.

c Kaplan-Meier.

### 3.3 Psychotropic drug outcomes

A total of 11.6 % of the patients with HF collected prescriptions of at least one of the outlined medications during one-year follow-up: Antidepressants (4.8 %), anxiolytics (3.0 %), hypnotics (5.0 %) or other sedative drugs (0.7 %). Among the controls, 2.4 % collected prescriptions of at least one of the abovementioned drugs, and the respective numbers were 1.1 %, 0.7 %, 1.0 %, and 0.1 %. The absolute risk of collecting one of the above outlined medications during follow-up standardized to the age, sex and selected comorbidity distributions of all included subjects was 9.8 % [95 % CI 9.6–10.0] for patients with HF versus 2.5 % [95 % CI 2.5–2.6] for controls. The corresponding relative risk was 3.85 [95 % CI 3.73–3.98]. When additionally standardizing for the distributions of highest achieved educational level as well as living alone status, the corresponding absolute risks were 8.3 % [95 % CI 8.2–8.5] versus 2.7 % [95 % CI 2.6–2.7], with a relative risk of 3.14 [95 % CI 3.05–3.23].

### 3.4 Psychiatric diagnosis outcomes

Among the patients with HF, 0.6 % received at least one of the outlined diagnoses during the first year after the HF diagnosis: depression (0.3 %), anxiety (0.1 %) or stress disorder (0.3 %). Among the controls, the corresponding numbers were significantly lower (overall <0.1 %). The absolute risk of being diagnosed with one of the outlined diagnoses during follow-up standardized to the age, sex and selected comorbidity distributions of all included subjects was 0.6 % [95 % CI 0.5–0.6] for patients with HF versus <0.1 % [95 % CI 0.0–0.1] for controls, corresponding to a relative risk of 12.90 [95 % CI 10.60–15.19]. When additionally standardizing for the distributions of highest achieved educational level as well as living alone status, the corresponding absolute risks were 0.5 % [95 % CI 0.4–0.5] versus <0.1 % [95 % CI 0.0–0.1], and a relative risk of 10.1 [95 % CI 8.26–11.89].

## 4 Discussion

In this nationwide register-based study of 94,712 patients with a first-time diagnosis of HF, nearly 12 % received either a registered diagnosis of depression, anxiety or stress disorders or started treatment with antidepressants, anxiolytics, hypnotics, or other sedative drugs during the first year of follow-up. This number was around five-fold higher compared to age- sex matched controls. Starting psychotropic medication accounted for most of this composite endpoint. The incidence of the composite endpoint was significantly higher among both patients diagnosed with HF during hospitalisation than in outpatient settings relative to controls. The current study supplements data from another nationwide study that looked at major depression and suicide among patients with newly diagnosed heart failure [1]. Previously, small prospective cohort studies had found that 21–22 % of patients had significant depression symptoms at a 1-year follow-up in HF patients without depression at discharge from a HF hospitalisation [12], [13]. Moreover, Lossnitzer et al. found symptoms of depression (minor and major) in 12.9 % of the cases at 1-year follow-up in HF patients without any signs of mood disorders at baseline [12].

In the present study, we tried to expand the perspective by looking both at development of depression but also anxiety or stress disorders as well as the medical treatment during the first year immediately after a diagnosis of heart failure. By using hospital-based registries to identify outcomes, we will miss some patients that had developed depression, anxiety, or stress disorders. Patients who are diagnosed solely by their general practitioner, or not diagnosed at all, will not be included, unless they were prescribed one of the included psychotropic drugs. We assume that it is primarily patients with milder degrees of these conditions that we have missed. This might explain why we found a lower incidence compared to what were reported from the abovementioned clinical studies, where questionnaires were used to evaluate depressive symptoms [12]. On the other hand, we also acknowledge that psychotropic medications are often prescribed to patients who do not have a relevant psychiatric diagnosis. This could be patients with distress, mild anxiety or sleep disorders that need sedatives for a period. They will appear in the registries as medicated but probably only having a transient need for psychotropic medication.

In the present study, patients with a prior diagnosis of depression, anxiety, or stress disorder within the last ten years prior to the HF diagnosis or prior use of psychotropic drugs within the last year were excluded from the study. This might also explain the lower outcome incidence in the present study, compared to other studies, since earlier depressive episodes are a known risk factor for recurrent depression both in HF patients and in elderly community subjects [12], [22], [23]. In the present study we were only interested in incident and not recurrent depression.

Dividing the HF patients in two subgroups (outpatients vs. hospitalized patients with first-time HF) documented that the incidence of the composite endpoint (HF patients vs controls) was not solely driven by the hospital admission per se. The cumulative incidence among the patients diagnosed with HF in outpatient clinics was much higher compared to controls. However, the incidence was even higher in patients diagnosed with HF during hospitalisation. This finding is probably well explained by the fact that the last group generally represents patients with more severe HF. However, we cannot exclude that detection/surveillance bias to some extent may be responsible for the difference between the two subgroups. But the rise of the curves separates from around day 10 to around the 4th month after the HF diagnosis, where most of the patients have been discharged for a long time.

A recent national cohort study also found a substantial number of patients with major depression and many suicides following a HF diagnosis [1]. So, to discover and handle these conditions, clinicians and HF specialist need be aware of the high incidence of mental problems that may lead to major depression and suicides in patients with HF [1], [10], [11]. This task is multidisciplinary and includes normal caretaking combined with screening questionnaires or clinical interviews [24]. The question is when the screening should be done to avoid under or overdiagnosis of mental issues in heart failure patients [24], [25].

Moreover, there does not seem to be an easy cure for anxiety and depressive symptoms in these patients. The SADHART CHF study did not find a cardiovascular or psychiatric benefit from treatment with sertraline a many study participants withdrew from the study due to side effects [26]. The same can be said about escitalopram [27]. Despite this, around 5 % of newly diagnosed heart failure patients were treated with an antidepressant during the first year after being diagnosed. The way forward may be non-pharmacologic and should include self-management patient education, physical exercises, and telehealth programs [28], [29]. However, heart failure specialists need to be aware that self-care systems may not apply for all [29].

### 4.1 Limitations

The present study has some limitations. As previously mentioned, we are not able to capture all patients with milder degrees of depression, anxiety, and stress disorders with the present study design. On the other hand, the register-based setup gives us a unique opportunity to accomplish a largescale, nationwide study with negligible “loss to follow up”. Furthermore, Kümler et al. demonstrated that the diagnosis of HF in the Danish National Patient Register holds a high a specificity (99 %) [30]. Thus, using the register enables accurate identification of patients with clinical HF as done in the present study.

Unfortunately, data according to income, level of education and status of living alone was missing for a not negligible percentage of the study population - especially among the controls. Thus, we performed analyses both with and without socioeconomic factors in the standardized average treatment effect multivariable Cox regression models. Results without the inclusion of socioeconomic factors were comparable to results including these factors.

## 5 Conclusion

Nearly 12 % of the patients with incident HF started treatment with psychotropic medication or received a registered first-time diagnosis with depression, anxiety, or stress disorder during the first year after being diagnosed with HF. This was around five-fold higher compared to the background population. Clinicians and HF specialist should be aware of this issue to detect and handle these conditions in the best way.

## Abbreviations

ATC: Anatomical Therapeutic Chemical
COPD: chronic obstructive pulmonary disease
HF: heart failure
HR: hazard ratio
ICD-8: 8th revision of the International Classification of Diseases
ICD-10: 10th revision of the International Classification of Diseases
ISCED: International Standard Classification of Education
NYHA: New York Heart association

## Availability of data and materials

In accordance with Statistics Denmark regulations, data cannot be shared or acquired. Researchers can apply for permission to access data on secure servers on Statistics Denmark.

## Funding

This work was supported by internal funding.

## CRediT authorship contribution statement

MWP, KK, NHA, PS and CTP have contributed substantially to the concept and design of the study. MWP, KK, MPA, GG and CTP contributed substantially to the acquisition and analysis of the data while MWP, KK, RR, MPA, MS, SJR, GG, SKLK, NHA and LK all contributed to the interpretation of the data. KK and NHA supervised the work. MWP, KK and NHA drafted the manuscript. KK, RR, MPA, MS, CP, SJR, GG, SLK, NHA, LK, PS and CTP revised the manuscript substantively for important intellectual content. All Authors have read and approved the final version of the submitted manuscript,

## Declaration of competing interest

The authors declare the following financial interests/personal relationships which may be considered as potential competing interests: MS was supported by a grant from the Novo Nordisk Foundation to the University of Copenhagen (NNF15SA0018404) and Helsefonden (20-B-0059).

CP reports receiving speaker fees from Lundbeck Pharma A/S, and research grants from the Danish Heart Foundation and the Eva and Henry Frænkel Memorial Foundation.

LK reports speaker fee from Novartis, Novo, AstraZeneca and Boehringer, unrelated to this topic.

CTP has received grants from Bayer and Sanofi unrelated to the current study.

KK reports speaker fees from Novartis.

MWP, RR, MPA, SJR, GG, SLK, NHA and PS declare that they have no competing interests.

## Appendix A Supplementary data

Supplementary tables Image 1

## References

[1] Crump C., Sundquist J., Kendler K.S., Sieh W., Edwards A.C., Sundquist K. (2022). Risks of depression and suicide after diagnosis with heart failure: a National Cohort Study. JACC Heart Fail..

[2] Sokoreli I., de Vries J.J.G., Pauws S.C., Steyerberg E.W. (2016). Depression and anxiety as predictors of mortality among heart failure patients: systematic review and meta-analysis. Heart Fail. Rev..

[3] Chobufo M.D., Khan S., Agbor V.N., Rahman E., Foryoung J.B., Jolayemi A. (2020). 10-year trend in the prevalence and predictors of depression among patients with heart failure in the USA from 2007–2016. Int. J. Cardiol..

[4] Jünger J., Schellberg D., Müller-Tasch T., Raupp G., Zugck C., Haunstetter A. (2005). Depression increasingly predicts mortality in the course of congestive heart failure. Eur. J. Heart Fail..

[5] Sokoreli I., de Vries J.J., Riistama J.M., Pauws S.C., Steyerberg E.W., Tesanovic A. (2016). Depression as an independent prognostic factor for all-cause mortality after a hospital admission for worsening heart failure. Int. J. Cardiol..

[6] Macchia A., Monte S., Pellegrini F., Pauws S.C., Steyerberg E.W., Tesanovic A. (2008). Depression worsens outcomes in elderly patients with heart failure: an analysis of 48,117 patients in a community setting. Eur. J. Heart Fail..

[7] Rasmussen A.A., Larsen S.H., Jensen M., Berg S.K., Rasmussen T.B., Borregaard B. (2021). Prognostic impact of self-reported health on clinical outcomes in patients with heart failure. Eur. Heart J. Qual. Care Clin. Outcomes.

[8] Vaccarino V., Kasl S.V., Abramson J., Krumholz H.M. (2001). Depressive symptoms and risk of functional decline and death in patients with heart failure. J. Am. Coll. Cardiol..

[9] Sbolli M., Fiuzat M., Cani D., O'Connor C.M. (2020). Depression and heart failure: the lonely comorbidity. Eur. J. Heart Fail..

[10] Ponikowski P., Voors A.A., Anker S.D., Bueno H., Cleland J.G., Coats A.J. (2016). 2016 ESC Guidelines for the diagnosis and treatment of acute and chronic heart failure: The Task Force for the diagnosis and treatment of acute and chronic heart failure of the European Society of Cardiology (ESC). Developed with the special contribution of the Heart Failure Association (HFA) of the ESC. Eur J Heart Fail..

[11] Havranek E.P., Spertus J.A., Masoudi F.A., Jones P.G., Rumsfeld J.S. (2004). Predictors of the onset of depressive symptoms in patients with heart failure. J. Am. Coll. Cardiol..

[12] Lossnitzer N., Herzog W., Stork S., Wild B., Muller-Tasch T., Lehmkuhl E. (2013). Incidence rates and predictors of major and minor depression in patients with heart failure. Int. J. Cardiol..

[13] Luijendijk H.J., Tiemeier H., van den Berg J.F., Bleumink G.S., Hofman A., Stricker B.H. (2010). Heart failure and incident late-life depression. J. Am. Geriatr. Soc..

[14] Pedersen C.B. (2011). The danish civil registration system. Scand. J. Public Health..

[15] Lynge E., Sandegaard J.L., Rebolj M. (2011). The Danish National Patient Register. Scand. J. Public Health.

[16] Kildemoes H.W., Sorensen H.T., Hallas J. (2011). The Danish National Prescription Registry. Scand. J. Public Health.

[17] Helweg-Larsen K. (2011). The danish register of causes of death. Scand. J. Public Health.

[18] Mors O., Perto G.P., Mortensen P.B. (2011). The danish psychiatric central research register. Scand. J. Public Health.

[19] Jensen V.M., Rasmussen A.W. (2011). Danish education registers. Scand. J. Public Health.

[20] Baadsgaard M., Quitzau J. (2011). Danish registers on personal income and transfer payments. Scand. J. Public Health.

[21] Andersen M.P., Valeri L., Starkopf L., Mortensen R.N., Sessa M., Kragholm K.H. (2019). The mediating effect of pupils' physical fitness on the relationship between family socioeconomic status and academic achievement in a Danish School Cohort. Sports Med..

[22] Freedland K.E., Rich M.W., Skala J.A., Carney R.M., Davila-Roman V.G., Jaffe A.S. (2003). Prevalence of depression in hospitalized patients with congestive heart failure. Psychosom. Med..

[23] Cole M.G., Dendukuri N. (2003). Risk factors for depression among elderly community subjects: a systematic review and meta-analysis. Am. J. Psychiatry.

[24] Jha M.K., Qamar A., Vaduganathan M., Charney D.S., Murrough J.W. (2019). Screening and management of depression in patients with cardiovascular disease: JACC state-of-the-art review. J Am Coll Cardiol..

[25] Gaffey A.E., Cavanagh C.E., Rosman L., Wang K., Deng Y., Sims M. (2022). Depressive symptoms and incident heart failure in the Jackson Heart Study: differential risk among Black Men and Women. J Am Heart Assoc.

[26] O’Connor C.M., Jiang W., Kuchibhatla M., Silva S.G., Cuffe M.S., Callwood D.D. (2010). Safety and efficacy of sertraline for depression in patients with heart failure: results of the SADHART-CHF (Sertraline against depression and heart disease in chronic heart Failure) trial. J. Am. Coll. Cardiol..

[27] Angermann C.E., Gelbrich G., Störk S., Gunold H., Edelmann F., Wachter R. (2016). Effect of escitalopram on all-cause mortality and hospitalization in patients with heart failure and depression: the MOOD-HF randomized clinical trial. JAMA.

[28] Blumenthal J.A., Babyak M.A., O’Connor C., Keteyian S., Landzberg J., Howlett J. (2012). Effects of exercise training on depressive symptoms in patients with chronic heart failure: the HF-action randomized trial. JAMA.

[29] Freedland K.E., Skala J.A., Steinmeyer B.C., Carney R.M., Rich M.W. (2021). Effects of depression on heart failure self-care. J. Card. Fail..

[30] Kumler T., Gislason G.H., Kirk V., Bay M., Nielsen O.W., Kober L. (2008). Accuracy of a heart failure diagnosis in administrative registers. Eur. J. Heart Fail..

