# Supplementary material for: One-year incidence of depression, anxiety, or stress disorders following a first-time heart failure diagnosis: A Danish nationwide registry-based study
Source: Am Heart J Plus. 2022 Dec 8;25:100240. doi: 10.1016/j.ahjo.2022.100240 (PMC10945984; doi:10.1016/j.ahjo.2022.100240)
Supplement: Supplementary file 1 — Supplementary tables [file mmc1.docx]

**Supplementary Material**

**Table S1**. **ICD-10 and ICD-8 codes for heart failure diagnosis according to inclusion**

| **ICD-10*** | DI50, DI420, DI426, DI427, DI428, DI429, DI110, DI130, DI1329 |
| --- | --- |
| **ICD-8**** | 425, 428, 4270, 4271, 42599, 42709, 42710, 42711, 42719, 42899 |

* used since 1994

** used 1971 - 1993

**Table S2**. **ICD-10 codes used to exclude patients with a preexisting psychiatric diagnosis.**

| **ICD-10** | DF00 – DF99: Mental or behavioral disorders |
| --- | --- |
| **Exception:** | DF1: Mental or behavioral disorders due to psychoactive drugs |

**Table S3**. **ATC codes used to exclude patients with preexisting use of psychoactive drugs.**

| **Antipsychotic drugs** | N05A |
| --- | --- |
| **Antidepressants** | N06A |
| **Anxiolytics** | N05B |
| **Hypnotic - or sedative drugs** | N05CD, N05CF, N05CH |

**Table S4**. **ICD-10 codes and ATC codes used to define preexisting comorbidity.**

| **Acute myocardial Infarction** | **ICD-10**: DI21. DI23 |
| --- | --- |
| **Ischemic heart disease** | **ICD-10**: DI20, DI21, DI23, DI24, DI25 |
| **Chronic kidney disease** | **ICD-10**: DN03, DN04, DN18, DN19, DE102, DE112, DE142,  DI12, DI13, DZ992 |
| **Stroke** | **ICD-10**: DI61, DI63, DI64, DI69, DI694 |
| **Peripherial artery disease** | **ICD-10**: DI70, DI74, DI739, DR02 |
| **Diabetes mellitus** | **ICD-10**: DE10, DE11, DE12, DE13, DE14  **ATC***: A10 |
| **Chronic obstructive pulmonary disease** | **ICD-10**: DJ44  **ATC****: R03BB, R03AL |
| **Hypertension** | **ICD-10**: DI10, DI11, DI12, DI13, DI15  **ATC*****: C02A, C02B, C02C, C02DA, C02DB, C02DD,  C02DG, C02L, C03A, C03B, C03D, C03E,  C03X, C07A, C07B, C07C, C07D, C07F, C08,  C08G, C09AA, C09BA, C09BB, C09CA,  C09DA, C09DB, C09XA02, C09XA52 |

* redemption of an antidiabetic drug during the last 180 days prior to the heart failure diagnosis

** redemption of medication for chronic obstructive pulmonary disease during the last 180 days prior to the heart failure diagnosis

*** redemption of ≥ 2 different antihypertensive drugs in 2 consecutive quarters during a 10-year period prior to the heart failure diagnosis

**Table S5**. **ICD-10 codes and ATC codes used according to outcome**

| **Depression** | **ICD-10**: DF32 |
| --- | --- |
| **Anxiety** | **ICD-10**: DF41 |
| **Stress disorder** | **ICD-10**: DF43 |
| **Antidepressants** | **ATC**: N06A |
| **Axiolytics** | **ATC**: N05B |
| **Hypnotics** | **ATC**: N055CD, N05CF, N05CH |
| **Other sedative drugs** | **ATC**: N05AA02, N05AF03, N05AH04 |
